# Gene Expression Dynamics in Rice Peduncles at the Heading Stage

**DOI:** 10.3389/fgene.2020.584678

**Published:** 2020-12-03

**Authors:** Manu Kandpal, Chandrapal Vishwakarma, Kushagra Krishnan, Viswanathan Chinnusamy, Ashwani Pareek, Manoj K. Sharma, Rita Sharma

**Affiliations:** ^1^Grass Genetics and Informatics Group, School of Computational and Integrative Sciences, Jawaharlal Nehru University, New Delhi, India; ^2^Division of Plant Physiology, ICAR-Indian Agricultural Research Institute, New Delhi, India; ^3^School of Life Sciences, Jawaharlal Nehru University, New Delhi, India; ^4^Grass Genetics and Informatics Group, School of Biotechnology, Jawaharlal Nehru University, New Delhi, India

**Keywords:** heading, rice, peduncle, stem, anther, panicle exsertion, internode

## Abstract

Improving grain yield in the staple food crop rice has been long sought goal of plant biotechnology. One of the traits with significant impact on rice breeding programs is peduncle elongation at the time of heading failing which leads to significant reduction in grain yield due to incomplete panicle exsertion. To decipher transcriptional dynamics and molecular players underlying peduncle elongation, we performed RNA sequencing analysis of elongating and non-elongating peduncles in two Indian cultivars, Swarna and Pokkali, at the time of heading. Along with genes associated with cell division and cell wall biosynthesis, we observed significant enrichment of genes associated with auxins, gibberellins, and brassinosteroid biosynthesis/signaling in the elongating peduncles before heading in both the genotypes. Similarly, genes associated with carbohydrate metabolism and mobilization, abiotic stress response along with cytokinin, abscisic acid, jasmonic acid, and ethylene biosynthesis/signaling were enriched in non-elongating peduncles post heading. Significant enrichment of genes belonging to key transcription factor families highlights their specialized roles in peduncle elongation and grain filling before and after heading, respectively. A comparison with anther/pollen development-related genes provided 76 candidates with overlapping roles in anther/pollen development and peduncle elongation. Some of these are important for carbohydrate remobilization to the developing grains. These can be engineered to combat with incomplete panicle exsertion in male sterile lines and manipulate carbohydrate dynamics in grasses. Overall, this study provides baseline information about potential target genes for engineering peduncle elongation with implications on plant height, biomass composition and grain yields in rice.

## Introduction

With huge diversity in morphological architectures of flowering plants, a thorough understanding of plant morphology is not only essential to appreciate the diverse shapes and forms exhibited by the plant world but also vital to engineering crop plants with improved agricultural traits. A slight modification in plant architecture can have a profound effect on the agronomic performance of crop plants. This was clearly demonstrated during the green revolution, where breeding for semi-dwarf genotypes led to higher yields and lodging resistance in rice (Coyne, 1980). Subsequently, several architectural features such as shape, size and angle of the leaf, number of tillers, and branching, were found to dramatically impact grain yield and stress responses (Peng et al., 2008).

One of the crucial morphological traits that significantly influences grain yield in rice is the elongation of the uppermost internode, also known as the peduncle. Elongation of peduncle at the time of heading facilitates the emergence of panicle from the flag leaf sheath, commonly known as panicle exsertion, which is also crucial for anther dehiscence and pollination (Bardenas, 1965). Incomplete panicle exsertion dramatically reduces seed set in rice and thus adversely affects agronomic yield (Yin et al., 2007). Moreover, peduncle elongation is highly sensitive to environmental factors. Several abiotic stress factors, particularly extremes of temperature and drought stress at the time of heading, result in dramatic yield losses by inhibiting peduncle elongation and, subsequent panicle exsertion and anther dehiscence in rice (Muthurajan et al., 2011; He and Serraj, 2012; Wu et al., 2016). Incomplete panicle exsertion is also observed in cytoplasmic male sterile lines with about 30–40% of panicles remaining enclosed in the flag leaf sheath, therefore making the spikelets unavailable for cross-pollination (Devi et al., 2011). Thus, incomplete panicle exsertion is one of the major impediments to obtain high yields in rice breeding programs. One of the common strategies used to combat this challenge involve spray of gibberellins but it significantly enhances the cost of production (Yin et al., 2007).

Furthermore, peduncle also contributes to grain filling. The excess photoassimilates, accumulated in the form of sucrose and starch in rice stems before heading, are mobilized to the panicles after heading to supplement grain filling (Chen and Wang, 2008; Wang et al., 2016). The transport of these non-structural carbohydrates from flag leaf to the panicle is facilitated through a continuous mature transport phloem network in leaf sheath, peduncle, and the rachis that supports spikelets on panicle (Slewinski, 2012). Since peduncle goes through a sink to source transition during the heading stage, elucidating transcriptional dynamics of peduncle at the time of heading is of fundamental importance to understand the mechanism of non-structural carbohydrate remobilization as well.

Till date, a handful of genes have been experimentally characterized to affect internode elongation through forward and/or reverse genetic strategies (Rutger and Carnahan, 1981; Luo et al., 2006; Zhu et al., 2011; Duan et al., 2012; Gao et al., 2012; Magome et al., 2013; Ji et al., 2014, 2019; Wang et al., 2017; Xie et al., 2018; Chu et al., 2019). However, lack of a comprehensive study to understand the overall gene expression dynamics during panicle elongation at the time heading is still limiting our understanding of panicle exsertion process in rice.

To fill this gap in our understanding, we investigated the transcriptional dynamics during peduncle elongation at the time of heading in two *indica* genotypes of rice, Swarna and Pokkali, so that genotype-specific effects could be minimized. These genotypes exhibit contrasting physiological and morphological traits in terms of cell wall composition, plant height, and grain yields (Jahn et al., 2011). Swarna, an elite *indica* cultivar, developed by crossing Vasista and Mahsuri rice varieties in 1982, has low glycemic index and is the most widely grown rice cultivar in Southern India (Rathinasabapathi et al., 2015). Pokkali, on the other hand, is a salinity tolerant *indica* landrace, mainly grown in coastal region of Southern India, for its salinity tolerance, high protein content, extra-large grains, peculiar taste, and medicinal properties (Hoang et al., 2016; Mishra et al., 2020). Expression profiling of elongating and non-elongating peduncles collected before and after heading, respectively, from both the genotypes, revealed conserved genetic elements and pathways underlying peduncle elongation at the time of heading in rice. These genes can be targeted using reverse genetic approaches to increase yield stability and stress tolerance in rice (Slewinski, 2012). Those implicated in cell wall biosynthesis would be important candidates for engineering stem biomass composition to enhance biofuel production. At the same time, genes regulating anther development as well as peduncle elongation can be leveraged to devise strategies to combat panicle enclosure in male sterile lines.

## Materials and Methods

### Plant Material and Sample Preparation

**FIGURE 1:**
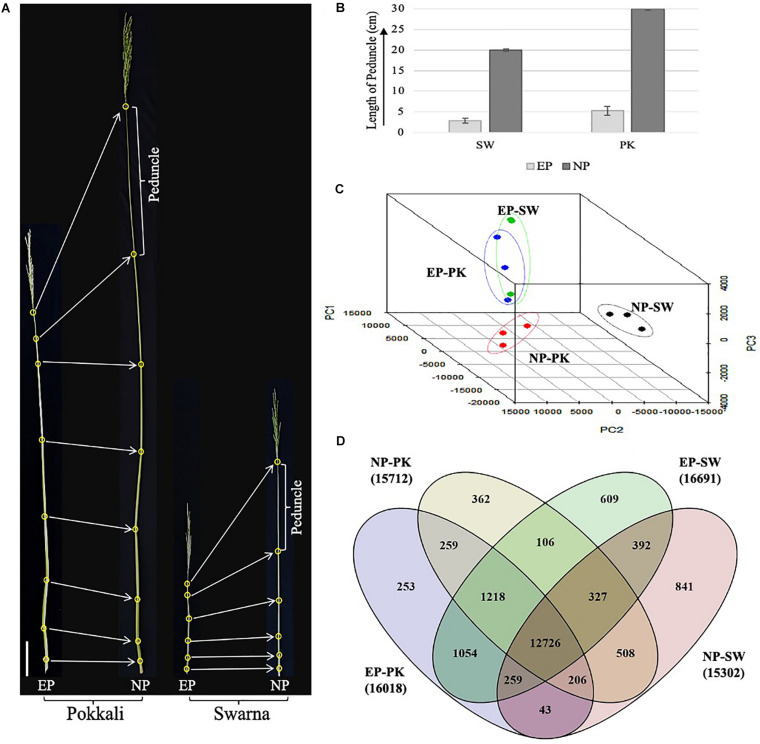
Phenotypic assessment and transcriptomic analysis of rice peduncles. **(A)** The topmost internode, also known as peduncle, is marked in both before heading and after heading Pokkali and Swarna plants. All leaves were removed to clearly show the stem internodes. **(B)** Graph showing comparative length of elongating and non-elongating peduncles in Swarna and Pokkali plants. **(C)** Principal component analysis of all three biological replicates of each sample. Dots of the same color represent biological replicates from same sample. **(D)** Venn diagram showing overlap between number of genes expressing with FPKM ≥ 1 in each sample. The total number of genes expressed in each sample is given in brackets with sample names. SW, Swarna; PK, Pokkali; EP, elongating peduncles before heading, and NP, non-elongating peduncles after heading.

*Oryza sativa* ssp. *indica* cultivars Swarna and Pokkali plants were grown in the fields under puddle transplanted conditions at ICAR-Indian Agricultural Research Institute (ICAR-IARI), Pusa, New Delhi, India. The phenotypic data on plant height, peduncle length, days to heading, and maturity were recorded from the field-grown plants. Elongating peduncles (EP) about 2–4 days before heading, and non-elongating peduncles (NP), about 2–4 days after heading, were separately sampled for each cultivar. Before heading, samples were collected when the maximum bulge could be observed in the flag leaf with the panicle still concealed beneath the leaf sheath. Conversely, heading stage samples were collected once the panicle had fully emerged out from the flag leaf sheath accompanied by flowering (Figure 1A). The samples were frozen in liquid nitrogen and stored at −80°C until further processing.

### RNA Isolation, Library Preparation, and Sequencing

Total RNA from three biological replicates of peduncle samples, collected from each cultivar at both elongating and non-elongating stages, was extracted using TRIzol^TM^ reagent (Invitrogen) as per the manufacturer’s instructions. The quantity and quality of RNA samples were determined using Nanodrop 2000 and Agilent 2100 Bioanalyzer. RNA samples with RIN value >7 were processed for library preparation using TruSeq^®^ Stranded Total RNA Sample Preparation Kit. Sequencing was performed on Hisequation 2000 with a read length of 100 bp (Illumina). The data has been submitted to NCBI GEO (Accession No. GSE157727).

### Data Analysis

After sequencing, raw reads were filtered to remove low-quality and adapter-contaminated reads using NGSQC Toolkit with default parameters (Patel and Jain, 2012). The high-quality reads with Phred score ≥30 were aligned with the rice genome available at Rice Genome Annotation Project Database version 7^1^, using Hisat version 2.1.0 with the option – rna-strandness RF, specific for stranded reads (Kim et al., 2015). Assembly was performed using Stringtie with −rf option (Pertea et al., 2015). The final assembly for all samples was obtained by Cuffmerge (Trapnell et al., 2012). Differential gene expression between both the samples was determined by Cuffdiff using the – fr option (Trapnell et al., 2012). The transcripts with log_2_ fold change ≥1 (upregulated genes) or ≤−1 (downregulated genes) with *P*-value cutoff ≤0.05 were considered differentially expressed. The expression levels of novel and annotated genes were quantified in terms of Fragments Per Kilobase per Million (FPKM) using Cufflinks (Trapnell et al., 2010). Principal component analysis (PCA) was performed on normalized FPKM values using prcomp function (center = TRUE, scale = FALSE) of the R package stats^2^. The 3D diagram of PCA for all 12 samples was plotted using the R package scatterplot3d (version 0.3–37^3^). Gene ontology (GO) enrichment for differentially expressed transcripts was performed using PlantGSEA^4^; (Yi et al., 2013), and *P*-values for enrichment analysis of each GO term were calculated using F-test. Only GO terms exhibiting *P*-value ≤0.05 were considered significant.

For metabolic pathway analysis, differentially expressed genes were also mapped on Mapman software using mapping file available for IRGSP loci (Thimm et al., 2004;^5^). The information about previously characterized genes for their roles in internode elongation, anther/pollen development, and grain filling was collected from OGRO (Overview of functionally characterized Genes in Rice Online database; Yamamoto et al., 2012;^6^) and FunRice genes (Yao et al., 2018;^7^) databases supplemented with manual literature survey. The list of differentially expressed genes in nine rice male sterile mutants was extracted from supplementary data provided by Lin et al. (2017).

## Results

### Plant Growth and Phenotypic Analysis

The plant height and developmental stages of *Oryza sativa spp. indica* genotypes, Swarna and Pokkali, grown in natural fields, were monitored over time especially closer to the heading stage. Both heading and maturity take longer in Pokkali as compared to Swarna plants as Pokkali plants took on an average of 123 and 151 days after sowing for heading and maturity, respectively, whereas, in Swarna plants, the average numbers of days for heading and maturity were 112 and 136 days after sowing, respectively. Phenotypic data collected from field-grown plants revealed striking contrast in plant height and peduncle length in both the genotypes (Figure 1A). The average height of Pokkali was almost double (158 cm) compared to Swarna (82 cm) plants. A similar trend was observed in the peduncle length. The average length of elongating peduncles 2–4 days before heading was 2.9 and 5.3 cm in Swarna and Pokkali, respectively. However, with prolific elongation observed closer to heading, peduncle lengths increased to an average of ∼20 cm in Swarna and ∼30 cm in Pokkali plants within 2–4 days post heading (Figure 1B). No significant increase in peduncle length was observed 4 days post heading, suggesting that the peduncle ceases to elongate as soon as the whole panicle has emerged out of the flag leaf.

### Sequencing Statistics and Principal Component Analysis

RNA sequencing of 12 libraries representing elongating (EP) and non-elongating peduncles (NP) of both the genotypes yielded a total of 690 million paired-end reads with an average read length of 100 bp. After removing low-quality and adapter contaminated reads, >616 million high-quality reads with an average Phred quality score (Q) ≥30 were used for reference-based transcriptome assembly. More than 90% of the high-quality reads from each replicate could be mapped onto the rice genome (Supplementary Table 1), and a total of 54,966 transcripts were annotated from both the cultivars.

To emphasize variation and correlation among EP and NP stages collected from both the genotypes, we performed PCA. As expected, elongating peduncles exhibited a distinct separation from the non-elongating ones (Figure 1C). Furthermore, the results revealed a higher correlation among EP samples of both the cultivars. In contrast, non-elongating peduncles from both the cultivars were well-separated indicating genotypic differences in the transcriptional repertoire of non-elongating peduncles (Figure 1C).

Further, genes expressed with FPKM ≥ 1 in all four samples were compared (Figure 1D). The total number of genes expressing in EP and NP stages in both the genotypes were comparable with more than 16,000 genes expressing at EP stage and over 15,000 genes expressing in NP samples from both the genotypes. A total of 12,726 transcripts were common to both EP and NP samples. In both the genotypes, Swarna had higher number of genes expressing exclusively in elongating (609) as well as non-elongating (841) peduncles (Figure 1D).

### *In silico* Validation of Gene Expression Patterns

**FIGURE 2:**
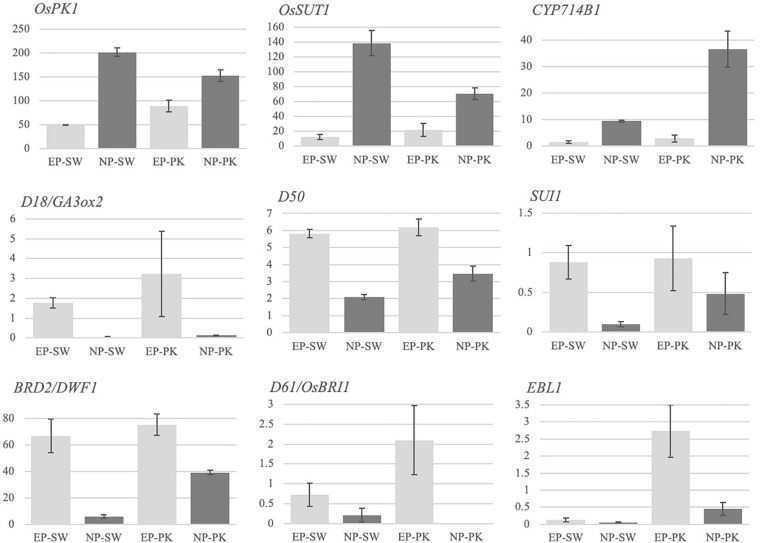
Expression profiles of previously characterized genes for involvement in peduncle elongation and/or panicle exsertion. The *Y*-axis represents FPKM values, and the *X*-axis represents samples. Error bars represent standard error between biological replicates. SW, Swarna; PK, Pokkali; EP, elongating peduncles, and NP, non-elongating peduncles. The names of the genes are given in italics on the top left of each bar graph.

Several genes in rice have been previously implicated in peduncle elongation and panicle exsertion at the time of heading using forward and/or reverse genetic approaches. To investigate if we could capture their differential accumulation during peduncle elongation, we checked the expression patterns of nine previously characterized genes in our data (Figure 2). Among these, *OsPK1* encoding a pyruvate kinase has been previously implicated in regulating plant height, panicle exsertion and carbohydrate transport during grain filling (Zhang et al., 2012). The mutant exhibits dwarfism, panicle enclosure, and reduced seed set. Consistent with its demonstrated role, *OsPK1* expressed at high levels in elongating peduncles before heading which escalated further in non-elongating peduncles post heading in both the genotypes (Figure 2). Another gene, *OsSUT1*, encoding the sucrose transporter, has been previously shown to express in rice peduncle with a critical role in facilitating the transport of assimilates from the flag leaf blade to the base of filling grains (Scofield et al., 2002, 2007). We observed a significant increase in *OsSUT1* expression post-heading in peduncles of both the genotypes (Figure 2).

A cytochrome P450 gene, *CYP714B1*, that encodes gibberellin 13-oxidase has been shown to reduce gibberellin (GA) activity in rice (Magome et al., 2013). Double mutation in CYP714B1 and *CYP714B2* resulted in elongation of uppermost internode in rice at the time of heading indicating their role in suppressing internode elongation post heading by downregulating GA activity. Conforming to these observations, *CYP714B1*, with negligible expression before heading, showed high expression in rice peduncles after heading in both the rice genotypes (Figure 2), whereas expression of a GA biosynthetic gene, *GA3Ox2* (*Gibberellin 3 beta-dioxygenase 2*) that plays a decisive role in internode elongation in rice, was detected in elongating peduncles pre-heading in both the genotypes (Iwamoto et al., 2011; Figure 2).

*Dwarfism 50* (*D50*) encoding inositol polyphosphate 5-phosphatase is essential for proper formation of intercalary meristem by regulating the direction of cell division, deposition of cell wall pectin, and actin organization (Sato-Izawa et al., 2012). As expected, *D50* exhibited higher expression in rice peduncles before heading with a significant decline observed post heading in both the genotypes (Figure 2). Similarly, another gene, *SUI1* (*Shortened Uppermost Internode 1*) encoding phosphatidylserine synthase, mediates cell expansion during elongation of uppermost internode in rice by regulating the secretion of cell wall components (Zhu et al., 2011; Yin et al., 2013; Ma et al., 2016). The *sui1* mutants exhibit immensely shortened uppermost internode and a sheathed panicle indicating its role in peduncle elongation and panicle exsertion at the time of heading. *SUI1*, though expressed at very low levels, exhibited a significant decline in expression post heading in both the genotypes (Figure 2).

Furthermore, rice genes associated with brassinosteroid biosynthesis and signaling have also been shown to affect internode elongation. Loss of function of *BRD2* (*Brassinosteroid-deficient dwarf 2*) involved in the brassinosteroid biosynthetic pathway and a putative BR receptor kinase, *BRI1* (*Brassinosteroid Insensitive 1*), leads to dwarf phenotypes confirming their role in internode elongation (Yamamuro et al., 2000; Hong et al., 2005). Both the genes exhibited a higher expression in elongating peduncles before heading in rice genotypes with a significant decline post heading in our data. Similarly, phytochrome-regulated *OsEREBP1*-like transcription factor *EBL1* which regulates internode elongation at the heading stage in rice by upregulating the expression of *ACO1* (*aminocyclopropane-1-carboxylate oxidase*), an enzyme associated with ethylene biosynthesis (Iwamoto and Takano, 2011), exhibited a significant decline in expression in Pokkali plants post heading (Figure 2). On the contrary, *EBL1* expression in Swarna plants was negligible both before and after heading. The differential expression of *EBL1* could be due to differential photoperiod sensitivity or response to ethylene levels in both the genotypes. Overall, we obtained the expected expression patterns of the previously characterized genes conforming to their demonstrated functions.

### Differential Gene Expression Analysis and Pathway Enrichment

**FIGURE 3:**
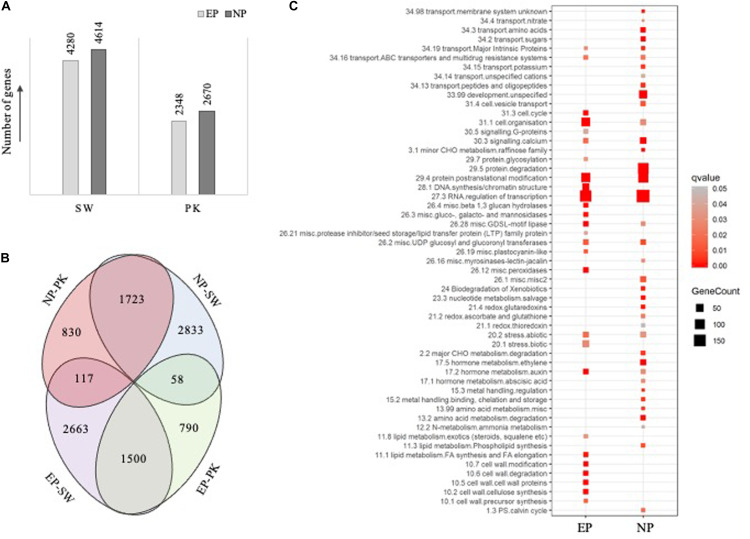
Results of differential expression analysis and pathway mapping. **(A)** Bar diagram showing the number of genes differentially expressed (fold change ≥ 2 and *P*-value ≤ 0.05) at the time of heading with EP representing the number of genes exhibiting higher expression in elongating peduncles before heading and NP representing number of genes exhibiting higher expression post heading. SW, Swarna; PK, Pokkali. **(B)** Venn diagrams showing the overlap between differentially expressed genes in both the genotypes. **(C)** Pathway enrichment analysis of the differentially expressed genes, with EP representing genes exhibiting higher expression in elongating peduncles before heading and NP representing genes exhibiting higher expression in non-elongating peduncles post heading, in both the genotypes. The size of square correlates with number of genes in each category while color signifies *P*-values as shown by legend on the right.

To identify genes exhibiting differential accumulation during peduncle elongation at the time of heading in both the genotypes, we used a cutoff ≥2-fold change with *P*-value ≤0.05. Expression levels of 8,894 and 5,018 genes were altered in Swarna and Pokkali, respectively. A total of 4,280 and 2,348 genes were expressed at higher levels in elongating peduncles of Swarna and Pokkali, respectively (Figure 3A), whereas 4,614 and 2,670 genes expressed at higher levels in non-elongating peduncles of Swarna and Pokkali, respectively. Since our study aimed to identify the genes with conserved roles in peduncle elongation instead of genotype-specific differences, we compared the differentially expressed gene sets from both the genotypes. A total of 1,500 genes were expressed at higher levels in EPs of both the genotypes compared to NPs (Figure 3B). Similarly, 1,723 genes expressed at higher levels in non-elongating peduncles. A large number of genes exhibiting contrasting transcript profiles imply genotype-specific variations likely responsible for variation in cell wall, biomass composition, stress tolerance, and other physiological parameters (Figure 3B).

To identify critical genes and pathways underlying peduncle elongation, irrespective of the genotype, we performed pathway enrichment analysis with 1,500 and 1,723 genes exhibiting differential expression in peduncles in both the genotypes (Supplementary Table 2). Among 1,500 genes with higher expression in elongating peduncles, those involved in transcriptional regulation, posttranslational modifications, cell organization, DNA synthesis, cell wall biosynthesis, lipid metabolism, stress response, cell cycle, and auxin metabolism were particularly enriched (Figure 3C). On the other hand, the enriched categories among 1,723 genes exhibiting higher expression in non-elongating peduncles were transcriptional regulation, protein degradation, posttranslational modification, development, calcium signaling, stress response, amino acid metabolism, sugar transport, and ethylene metabolism (Figure 3C). These observations conform with the developmental events involving active cell division in the elongating peduncles and mobilization of carbohydrates in non-elongating peduncles after heading. Among those exclusively upregulated in elongating peduncles of Swarna, protein synthesis, RNA processing, development, and photosynthetic light reaction categories were enriched. On the contrary, genes associated with vesicular transport, signaling, and cell wall biosynthesis were exclusively enriched in elongating peduncles of Pokkali (Supplementary Table 5). Interestingly, genes associated with photosynthesis were exclusively upregulated in non-elongating peduncles of Pokkali, whereas those associated with protein degradation, posttranslational modification, signaling, lipid metabolism, and protein targeting were exclusively enriched in non-elongating peduncles of Swarna plants (Supplementary Table 5).

### In-Depth Analysis of Differentially Expressed Genes Associated With Transcriptional Regulation, Cell Wall, Carbohydrate, and Hormone Metabolism

Taking cues from the pathways enriched during peduncle elongation, we performed an in-depth profiling of differentially expressed genes associated with cell wall biosynthesis and modification, carbohydrate metabolism and transport, hormone biosynthesis, signaling, and response, and transcriptional regulation.

#### Cell Wall Biosynthesis and Modification

**FIGURE 4:**
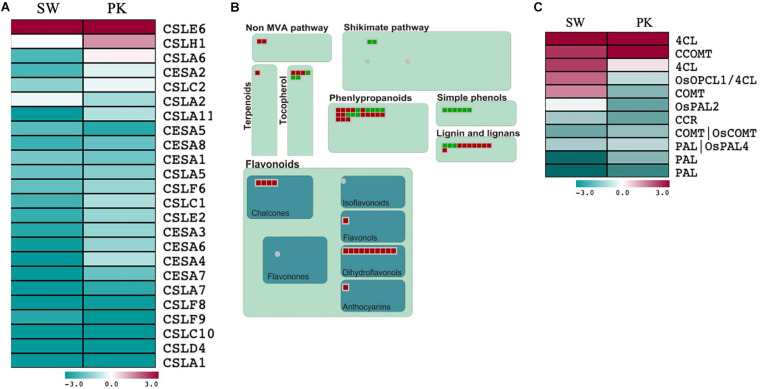
Expression profiling of genes associated with cell wall biosynthesis and secondary metabolism. **(A)** Heatmap showing differential expression of cell wall biosynthesis-related genes during heading in Swarna (SW) and Pokkali (PK) plants. Teal blue color represents genes exhibiting ≥2-fold expression in elongating peduncles while cayenne red indicates ≥2-fold expression in non-elongating peduncles post heading. Gene names are given on the right of the heatmap. **(B)** MapMan secondary metabolism overview with green boxes presenting genes with higher expression in elongating peduncles and red boxes representing genes exhibiting higher expression in non-elongating peduncles. **(C)** Heatmap showing differential expression of lignin biosynthesis-related genes. Teal blue color represents genes exhibiting ≥2-fold expression in elongating peduncles while cayenne red indicates ≥2-fold expression in non-elongating peduncles post heading. Gene names are given on the right of the heatmap.

A total of 24 cellulose synthases (*CESA*) and cellulose synthase-like (*CSL*) genes were differentially expressed in both the genotypes with 22 of them exhibiting higher expression in elongating peduncles compared to non-elongating ones (Figure 4A; Supplementary Table 3A). The CSL genes exhibiting higher expression in elongating peduncles belong to the CSLA, CSLC, CSLC, CSLD, and CSLF families (Figure 4A). *CSLE6* and *CSLH1*, on the contrary, exhibited higher expression post heading (Figure 4A). Several genes implicated in secondary metabolite biosynthesis also exhibited higher expression in post heading peduncles (Figure 4B).

However, genes associated with lignin biosynthesis showed a mixed pattern with *PAL* (phenylalanine ammonia-lyase) and *CCR* (cinnamoyl-CoA reductase) genes expressing at higher levels in EPs while *4CL* (4-coumarate: coenzyme A ligase) and *CCOMT* (caffeoyl CoA 3-O-methyltransferase) exhibited higher expression in non-elongating peduncles (Figure 4C; Supplementary Table 3B). Further, three genes encoding 4CLs (4-coumarate: coenzyme A ligases) and COMT (caffeic acid 3-O-methyltransferase) exhibited contrasting patterns in both the genotypes with higher expression in elongating peduncles of Pokkali and non-elongating peduncles of Swarna rice. These results may be explained by varying levels of lignification of peduncles after heading in both the genotypes.

#### Carbohydrate Metabolism and Transport

**FIGURE 5:**
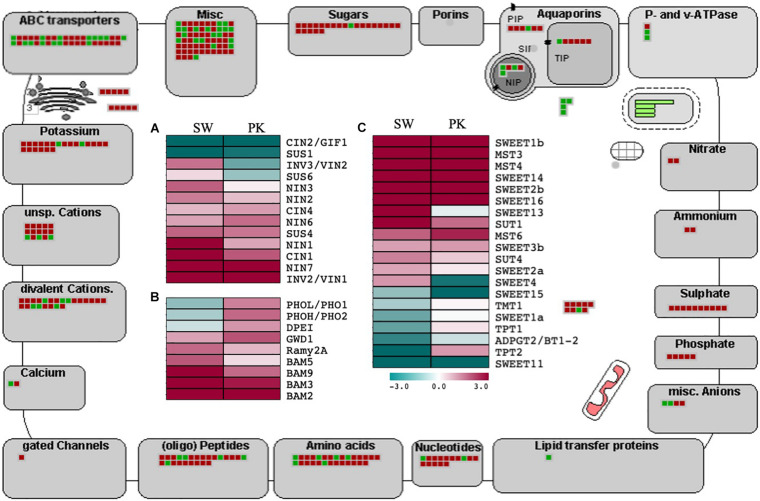
Expression profiling of genes associated with carbohydrate metabolism and transport. The outer illustration is the MapMan transport overview with green boxes presenting genes with higher expression in elongating peduncles and red boxes representing genes exhibiting higher expression in non-elongating peduncles. The inner portion presents heatmaps showing differential regulation of genes associated with sucrose metabolism **(A)**, starch metabolism **(B)**, and sugar transport **(C)** Swarna (SW) and Pokkali (PK) plants. Teal blue color represents ≥2 folds expression in elongating peduncles and cayenne red indicates ≥2 folds expression in non-elongating peduncles post heading. Gene names are given on the right of each heatmap.

Since the peduncle comprises an essential component of the transport system facilitating carbohydrate mobilization during grain filling post heading, we investigated the expression of rice genes implicated in sucrose and starch metabolism as well as transport in our data (Supplementary Table 3C). Several genes regulating sucrose and starch degradation, as well as sugar transport, exhibited differential expression in peduncles at the time of heading (Figure 5). Notable among those were sucrose synthases (Huber and Akazawa, 1986; Stein and Granot, 2019). Rice *SUS4* (*sucrose synthase 4*), which has previously been shown to be associated with grain filling exhibited higher expression in post heading peduncles, whereas *SUS1* (*sucrose synthase 1*) having a more ubiquitous role was downregulated after heading in peduncles (Figure 5A). Antagonistic patterns of these genes are likely due to their differential activity in different tissues. *SUS1* is implicated in the regulation of vegetative growth, while *SUS4* is involved in assimilate portioning to the caryopsis during grain filling (Sparks(ed.), 2012).

Invertases modulate the hexose to sucrose ratio by hydrolyzing sucrose into glucose and fructose. Most of the invertases exhibited a higher expression in post heading peduncles (Figure 5A) Both *cytosolic invertase 1* (*CIN1*) and vacuolar invertase (*INV3*) were upregulated after heading (Morey et al., 2018). A similar trend was observed for other starch metabolism and sugar transport-related genes with significant upregulation after heading (Figure 5B).

Before loading into the sieve element–companion cell complex through sucrose transporters, sucrose is effluxed from phloem parenchyma cells by *SWEET* (*Sugars Will Eventually Be Exported Transporters*) genes (Ayre, 2011; Chen et al., 2012). We observed significant upregulation of several *SWEET* genes in rice peduncles after heading (Figure 5C). Besides, the genes encoding ABC, potassium, calcium, peptides, amino acids, nucleotides, phosphate, sulfate, ammonium, nitrate, and *P*- and v-ATPase transporters also exhibited a higher expression post heading. Several genes associated with carbohydrate partitioning such as *SUT1* and *GWD1* also exhibited higher expression in peduncles after heading (Figure 5C). The sucrose transporter-encoding gene *OsSUT1* has previously been shown to play a role in long-distance transport of assimilates from the flag leaf blade to the base of filling grains through the flag leaf sheath and uppermost internode, referred to as peduncle in this study (Scofield et al., 2002, 2007). *GWD1*, encoding alpha-glucan water dikinase, has been implicated in carbohydrate partitioning after heading in rice (Wada et al., 2017). Loss of function of *GWD1* led to hyperaccumulation of starch in leaves (Hirose et al., 2013).

#### Hormone biosynthesis, signaling, and response

**FIGURE 6:**
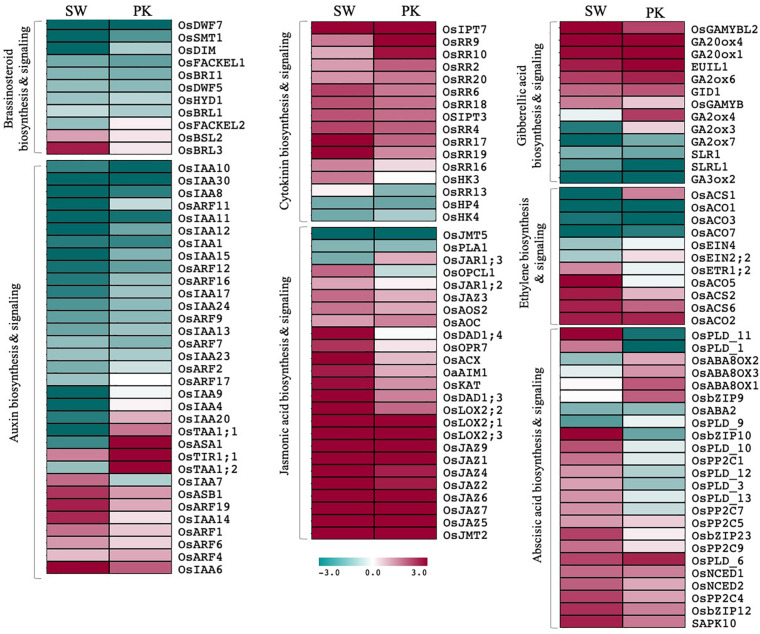
Expression profiling of genes associated with hormone biosynthesis and signaling. Heatmaps showing differential expression of genes associated with hormone biosynthesis and signaling in Swarna (SW) and Pokkali (PK). Teal blue color represents ≥2 folds expression in elongating peduncles and cayenne red indicates ≥2 folds expression in non-elongating peduncles post heading. Gene names are given on the right of the heatmaps and hormone categories are given on the left.

Among differentially expressed genes, 68 genes have been previously implicated in plant hormone biosynthesis/signaling (Figure 6; Supplementary Table 3D). Although some of them showed a contrasting pattern, by and large genes associated with auxins and brassinosteroids (BR) exhibited higher expression in elongating peduncles before heading, whereas those regulating abscisic acid (ABA), jasmonic acid (JA), and cytokinin metabolism were predominantly upregulated post heading (Figure 6). The genes associated with GA and ethylene biosynthesis/signaling and response exhibited a mixed pattern though overall trend suggested higher expression of larger number of genes post heading (Figure 6).

Similarly, although majority of ABA-biosynthesis/signaling genes expressed at higher levels in post-heading peduncles, some exhibited contrasting expression patterns in both genotypes. Several of the ABA signaling-related genes which exhibited a higher expression after heading in peduncles of Swarna, expressed at higher levels before heading in Pokkali plants (Figure 6). Conversely, some of the genes associated with ABA deactivation that expressed at higher levels before heading in Swarna were upregulated in peduncles of Pokkali. These observations point to ABA activation in Swarna while ABA deactivation in Pokkali after heading.

#### Transcription Factor-Encoding Genes

**FIGURE 7:**
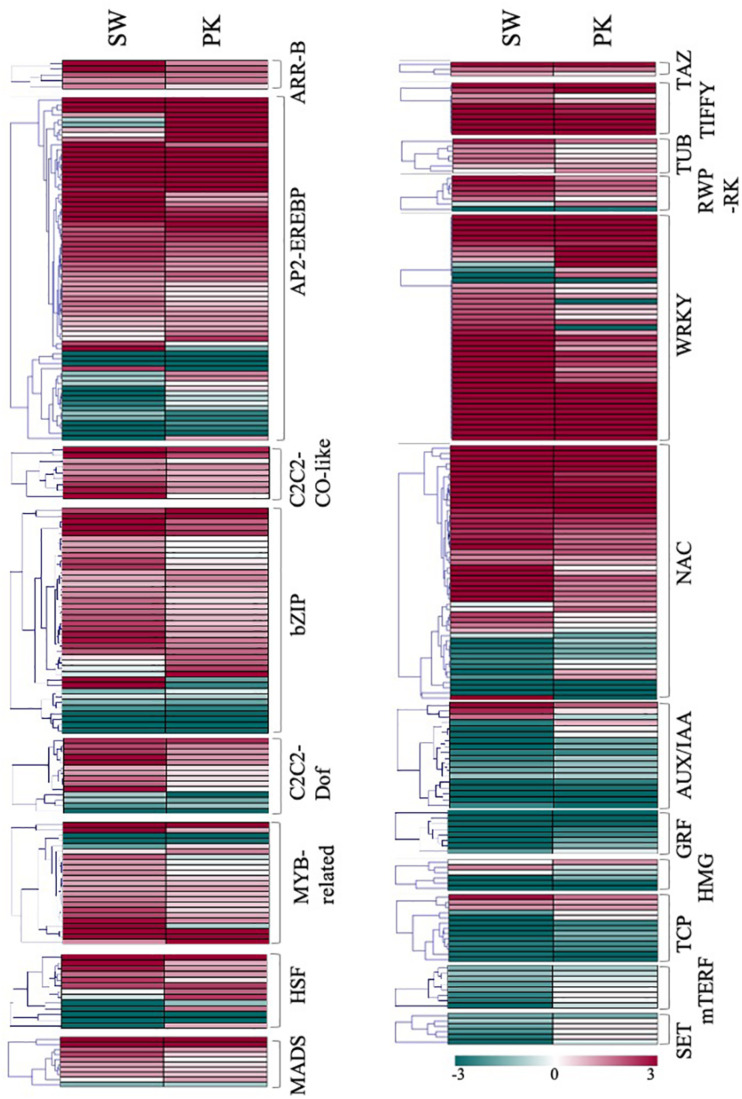
Expression profiling of transcription factor-encoding genes. Heatmaps showing differential expression of transcription factor-encoding genes in Swarna (SW) and Pokkali (PK). Members of the same gene family are clubbed together in one heatmap. Teal blue color represents ≥2 fold expression in elongating peduncles while cayenne red indicates ≥2 fold expression in non-elongating peduncles post heading. Names of the transcription factor families are given on the right of each heatmap.

A total of 836 transcription factors (TFs), belonging to more than twenty families, were differentially expressed in rice peduncles after heading (Supplementary Table 3E). Albeit members of the same transcription factor family do not necessarily perform the same function, we observed a large number of genes belonging to the same family exhibiting similar expression patterns, thereby indicating similar/coordinated functions of the gene family members. Genes belonging to ARF, AUX-IAA, GRF, HMG, OFP, Trihelix, PHD, and TCP families, mostly implicated in cell division and elongation, exhibited a higher expression in elongating peduncles (Figure 7), whereas those belonging to ARR-B, C2C2-CO like C2C2-Dof, MADS, TRAF, AP2-EREBP, bZIP, NAC, G2-like, HSF, OPHANS, WRKY, TIFFY, REP-RK, and PLATZ families expressed at higher levels post heading. Members of C3H, SNF2, C2H2, bHLH, GRAS, and HB exhibited mixed patterns (Figure 7).

### Differential Expression of Anther/Pollen-Associated Genes During Peduncle Elongation

**FIGURE 8:**
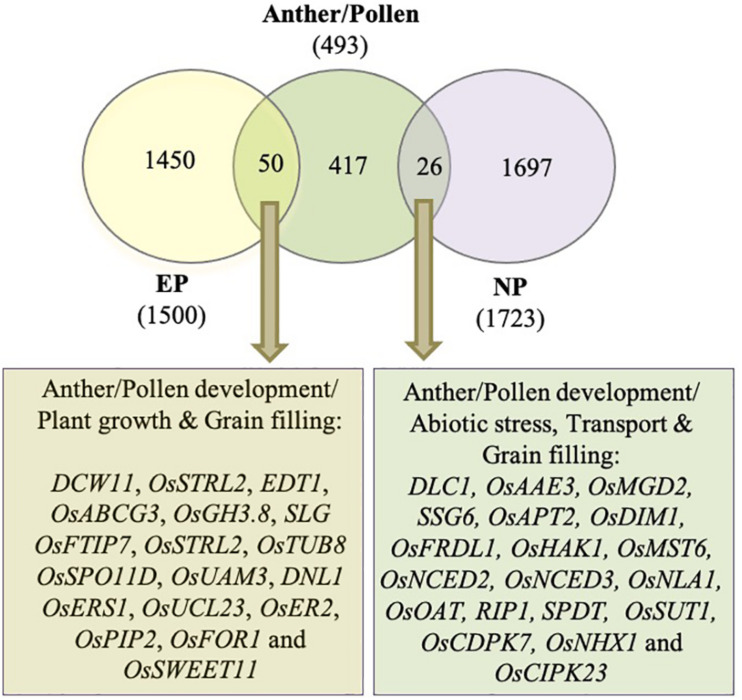
Overlap between differentially expressed genes during peduncle elongation and anther development. The Venn diagram shows the overlap between genes exhibiting ≥2 fold expression in elongating peduncles (EP), non-elongating peduncles (NP), and anther/pollen development. The key pathways and characterized genes highlighted in the gene set common between EP/NP and anther/pollen associated genes are given.

Incomplete peduncle elongation and panicle enclosure are frequently observed in male sterile lines. To investigate if the genes associated with peduncle elongation have anything to do with anther development, we compared the differentially expressed genes during peduncle elongation with those associated with anther/pollen development. Previously, Lin et al. (2017) had generated a rice gene co-expression network for anther development (RiceAntherNet) from 57 rice anther tissue microarrays. They mapped differentially expressed genes from nine rice male-sterile mutants onto this network and shortlisted a set of 286 genes associated with pollen formation. We appended this list with anther/pollen-associated genes extracted from Funrice and OGRO databases of rice. Finally, a list of 493 genes implicated in anther/pollen development in rice was compiled. Out of these 493 genes, 76 (15%) were differentially expressed in peduncles at the time of heading in both the genotypes (Figure 8; Supplementary Table 4). Out of these, 50 overlapped with genes exhibiting ≥2-fold expression in EPs, whereas 26 overlapped with genes exhibiting ≥2-fold expression in NPs (Figure 8). Most of the anther development-related genes that exhibited higher expression in elongating peduncles before heading (50 in number) encode glycoside hydrolases, serine carboxypeptidases, kinases, and other cell wall-related proteins. Some of these genes have already been characterized for their roles in regulating plant height, grain filling and/or anther/pollen development. Conversely, those overlapping with high-expressing genes in non-elongating peduncles (26 in number) as well as anther development have been implicated in abiotic stress and grain filling.

## Discussion

Peduncle elongation at the time of heading not only is crucial for panicle exsertion but also exerts significant impact on grain yield. To identify molecular players regulating peduncle elongation and associated agronomically important traits, we have performed RNA sequencing with peduncles before and after heading collected from two contrasting genotypes of rice. Although the total number of expressed genes (FPKM ≥1) was similar in both the genotypes, those exhibiting differential accumulation between EP and NP stages in Swarna were almost double in number compared to differentially expressed genes in Pokkali. This could possibly be because Swarna has to make more transcriptional adjustments to cope up with the abiotic stress post heading compared to the relatively stress-tolerant Pokkali. Further, most of the genes exclusively differentially expressed in Swarna are involved in the basic metabolic functions including protein synthesis, degradation and posttranslational modifications, signaling, RNA processing, lipid metabolism, transport, development, and abiotic stress response. PCA analysis further highlighted noticeable distance between non-elongating peduncles compared to elongating peduncles likely due to differences in post heading stem biomass composition and stress response of these genotypes (Jahn et al., 2011). However, when we compared the expression patterns of previously characterized genes which have been experimentally shown to regulate peduncle elongation and grain filling in rice, all of them exhibited a similar expression pattern in both the genotypes though the amplitude of expression and fold change varied, indicating that the genetic factors underlying peduncle elongation are conserved across genotypes. Furthermore, changes observed at the transcriptional level may not reflect in protein abundance. Earlier, comparative analysis of transcript and protein abundance of Pokkali and IR29 revealed a much higher number of genes differentially expressed in Pokkali at the protein level with little difference at the transcript level in response to salt stress (Li et al., 2018). In fact, 75.5% of genes exhibiting high protein abundance did not show relatively high transcriptional abundance in Pokkali. The authors suggested that higher stability and efficient loading of mRNAs in Pokkali could be the possible explanation for lower number of differentially expressed transcripts in Pokkali.

### Candidate Genes Associated With Peduncle Elongation and Grain Filling

Elongation of peduncles is accompanied by active cell division and cell wall biosynthesis. This is obvious from enrichment of glycosyltransferase (GTs) in elongating peduncles of both the genotypes. Glycosyltransferases comprise a large gene family with more than 600 genes in rice which have been classified into subfamilies based on the presence of distinct sequence domains (Cao et al., 2008). Among these, CESAs belonging to the GT2 subfamily are mainly involved in cellulose biosynthesis, while cellulose synthase-like (CSL) genes play key roles in hemicellulose biosynthesis (Richmond and Somerville, 2001). CSLAs form the β-1,4-mannan backbone of galactomannan and glucomannan, CSLDs determine cellulose and xylose content, whereas, CSLFs are essential for grass-specific mixed linkage glucan biosynthesis (Li et al., 2009; Luan et al., 2011; Vega-Sánchez et al., 2012; Kaur et al., 2017). Many of these have been shown to exhibit tissue and developmental stage-specific expression. In-depth analysis of these genes in our data provided 22 candidates with significant accumulation in EPs relative to NPs implying their potential role in regulating cell wall biosynthesis and biomass composition in elongating rice peduncles.

Also, a shift from primary to secondary cell wall biosynthesis is anticipated at the time of heading for providing mechanical strength required to support increase in panicle weight due to grain filling. *OsMYB103* has earlier been reported to play a role in secondary cell wall in sclerenchyma by activating *OsCESA4*, *OsCESA7*, *OsCESA9*, and *OsBC1* (Hirano et al., 2013; Yang et al., 2014; Ye et al., 2015). All of these genes exhibit higher accumulation in EPs indicating that molecular players responsible for secondary cell wall biosynthesis are initiated before heading. On the other hand, higher accumulation of genes associated with biosynthesis of secondary metabolites in NPs indicates their role in post-heading resistance to pathogens though some of them may also act as precursors for secondary cell wall components. Genes involved in lignin biosynthesis exhibited contrasting pattern of expression in Swarna and Pokkali likely due to varied lignin composition in these genotypes (Jahn et al., 2011).

Among the differentially expressed transcription factor families, members of MYB, NAC, WRKY, bZIP, PLATZ, and HSF transcription factors have been previously associated with secondary growth, abiotic stress response, and carbon mobilization (Oh et al., 2003; Agarwal et al., 2006; Zhang et al., 2010; Golldack et al., 2014). Further characterization of these genes would not only help to decipher the molecular mechanism underlying peduncle elongation and biomass composition but will also help in optimizing carbon remobilization in rice.

### Interplay of Phytohormones During Peduncle Elongation in Rice

Phytohormones play crucial roles in plant growth and development. Previous studies have shown the involvement of auxins, gibberellins, and brassinosteroids in promoting cell division and growth during internode elongation in rice (Chen, 2001; Yin et al., 2002; Tong et al., 2014; Oh et al., 2020). For instance, BR receptor kinase *OsBRI1* of rice positively regulates internode elongation by inducing the formation of the intercalary meristems (Yamamuro et al., 2000). Loss of function of this gene prevents internode elongation and bending of the lamina joint. We noticed a high expression of *OsBRI1* before heading in both the genotypes with an apparent decline after heading. Except for *OsBRL3*, which is known to play a role in BR perception in roots (Nakamura et al., 2006), all other brassinosteroid-related genes also exhibited a higher expression in elongating peduncles before heading. Similarly, auxins also act as a signal for cell elongation (Chen, 2001). Developing panicles in grasses export auxin to stem for promoting elongation (Wolbang et al., 2004). Except for a few, most of the auxin-related genes were expressed at higher levels in rice peduncles before heading. Among the genes exhibiting higher expression post heading, *OsARF19* is a negative regulator of cell division as its overexpression in rice leads to dwarf phenotype. Furthermore, *OsARF19* directly regulates the expression of BR receptor *OsBRI1* by binding to its regulatory region (Zhang et al., 2015). Upregulation of *OsARF19* and downregulation of *OsBRI1* after heading therefore suggest that *OsARF19* likely suppresses peduncle elongation after heading by downregulating *OsBRI1*.

Gibberellic acid (GA) has also been shown to act as a key determining factor in peduncle elongation and panicle exsertion (Yin et al., 2007; Chen et al., 2013). We observed a mixed pattern of the genes associated with GA and ethylene biosynthesis/signaling likely due to multilevel cross-talk or negative feedback regulation of some of them. GA biosynthetic gene, *gibberellin 3-beta-hydroxylase* (*GA3Ox*), which catalyzes the final step of GA biosynthesis in plants by converting GA20 to GA1, exhibited a higher expression before heading with notable downregulation after heading (Iwamoto et al., 2011; Figure 6). *OsGA3Ox2* corresponds to the *D18* mutant that exhibits severe dwarfism due to decrease in level of active GA1 (Itoh et al., 2002). Antisense plants with reduced *OsGA3Ox2* expression exhibited a higher expression of another gene *OsGA20Ox1* that regulates the synthesis of GA21 from GA53 (Itoh et al., 2002). Interestingly, both the genes in our study exhibited antagonistic patterns with *GA3Ox2* exhibiting downregulation and *GA20Ox1* showing upregulation after heading. Conversely, GA receptor *Gibberellin Insensitive Dwarf 1* (*GID1*) which makes a complex with GA and induces degradation of GA repressing DELLA proteins is upregulated (Ueguchi-Tanaka et al., 2007; Sun, 2008) while DELLA protein-encoding genes, *SLR1* (*SLENDER RICE 1*) and *SLR1 Like-1* (*SLRL1*), are downregulated after heading, indicating that suppression of GA activity after heading is not mediated through DELLA proteins. It has been shown that a high concentration of BR triggers BR-associated inactivation of GA. The BR signaling component involved in this inactivation process, *BRI1*-*GSK2*-*DLT*, is upregulated before heading in our data (Tong et al., 2014). The effect of this can be observed with increased expression of GA deactivation genes after heading. For instance, *CYP714B1* encoding gibberellin 13-oxidase which reduces gibberellin activity in rice as well as *GA2ox4* and *GA2ox6*, implicated in endogenous GA deactivation (Chu et al., 2019), expressed at higher levels in peduncles after heading (Lo et al., 2008; Magome et al., 2013). Overall, regulation of *OsBRI1* by *OsARF19* and its (*OsBRI1*) role in deactivation of GA indicates interaction of auxin, brassinosteroids, and gibberellins in maintaining the hormonal homeostasis during peduncle elongation (Zhang et al., 2015).

Cytokinins, on the other hand, have been associated with post-anthesis grain filling in rice (Yang et al., 2002; Zhang et al., 2009). Since mostly cytokinins are transported from roots to shoots and other aerial organs *via* xylem sap, elevated levels of cytokinins in rice peduncles after heading suggest their role in grain filling (Yang et al., 2002; Zhang et al., 2009). Panicle enclosure has been shown to adversely affect spikelet fertility and grain yield (He and Serraj, 2012). A genome-wide association study using 205 rice cultivars clearly drew a positive correlation between panicle exsertion and 1,000-Grain Weight (Zhan et al., 2019). The grain filling is facilitated by transport of carbohydrate resources stored in elongating stem in rice in the form of starch and sucrose (Scofield et al., 2007, 2009; Slewinski, 2012). The carbon remobilization from the stem reserves to grains possibly occurs by an apoplasmic mechanism through sugar transporters (Slewinski, 2012). Many of the sugar transport genes were upregulated in the peduncles suggesting their role in grain filling. For example, *OsSUT1*, a member of the sucrose transporter family, is required for actively pumping sucrose into phloem against the concentration gradient (Scofield et al., 2007). In the present study, its upregulation before heading conforms with its role in grain filling. Monosaccharide transporter genes, *OsMST4* and *6*, implicated in supplying monosaccharides for seed development during grain filling, are also upregulated in NPs after heading in our study (Wang et al., 2007, 2008). *OsSUS4*, involved in portioning of the carbon assimilates to the caryopsis during grain filling (Sparks(ed.), 2012), is also upregulated in peduncles after heading. This suggests that peduncle plays a critical role in transporting carbohydrate reserves stored during stem elongation to the developing grains, and hence, rice genes exhibiting increased expression in NPs after heading may be leveraged to optimize carbohydrate mobilization in rice.

Jasmonic acid (JA) is another important phytohormone associated with biotic and abiotic stress response and is known to inhibit shoot growth in rice explaining the upregulation of JA biosynthetic genes after heading (Liu et al., 2015). The level of JAs has also been associated with availability of carbon and soluble sugars, indicating the role of JA in carbon remobilization (Huang et al., 2017). Upregulation of ABA-related genes after heading has been associated with abiotic stress response during reproductive development (Baron et al., 2012; Singh et al., 2012). Some genes implicated in ABA biosynthesis and signaling exhibited contrasting patterns of expression in both the genotypes. Further investigations would be required to understand if this can be explained by differential stress tolerance in Swarna and Pokkali post heading. Overall, our data shows a clear trend with higher expression of growth hormones, viz., AUX, GA, and BR, in elongating peduncles before heading, whereas JA, ABA, and cytokinins, associated with stress response, sugar signaling, and remobilization, exhibit higher expression after heading.

### Common Genetic Elements Underlying Peduncle Elongation and Male Reproductive Organ Development

Crop improvement in rice to optimize agronomic yields and stress tolerance largely relies on breeding programs. However, panicle enclosure due to impeded peduncle elongation in male sterile lines is a major challenge in achieving these goals. A number of studies have associated enclosed spikelets with inhibition of anther dehiscence, decreased pollen viability, slower pollen tube growth, and abnormal ovary development (Ekanayake et al., 1989, 1990; Jagadish et al., 2011, 2015). A comparative study of cytoplasmic male sterile line with its maintainer lines during panicle development revealed increased levels of ABA and reduced levels of GA and IAA in the cytoplasmic male sterile line (Chang’en et al., 1998). Deficiency of IAA and GAs and excess of ABA hinder anther development and induce pollen abortion (Shimizu and Kuno, 1967; Nakajima et al., 1991; Sunohara et al., 2009). Later, Yin et al. (2007) showed that deficiency of indole-3-acetic acid in panicle of male sterile lines downregulates GA oxidase (*OsGA3ox2*) resulting in decreased active GA levels, thereby hampering peduncle elongation manifested in the form of enclosed panicles and poor seed set (Yin et al., 2007). In the present study, several genes related to anther and pollen development were differentially expressed in both elongating and non-elongating peduncles. Although it is a well-known fact that male sterile lines exhibit varying degrees of panicle enclosure, the genetic basis of the relationship between anther development and peduncle elongation has not been established till date. To identify the common genetic elements regulating peduncle elongation and anther/pollen development, we compiled a list of 493 genes implicated in anther/pollen development and checked their expression in peduncles.

Most of the anther development-related genes, also exhibiting higher expression in elongating peduncles before heading (50 in number), encode glycoside hydrolases, serine carboxypeptidases, and kinases. Some of these genes have already been characterized for their roles in anther development and/or internode elongation. Among these, *DCW11* (Fujii and Toriyama, 2008), *OsSTRL2* [strictosidine synthase; (Zou et al., 2017)], *EDT1* [*Earlier Degraded Tapetum 1*; (Bai et al., 2019)], *OsABCG3* [ATP-binding cassette transporter; (Chang et al., 2018)], *OsGH3.8* (Yadav et al., 2011), *OsSPO11D* (Shingu et al., 2012), *OsUAM3* (Sumiyoshi et al., 2015), and *OsUCL23* (Zhang et al., 2020) have been shown to regulate diverse aspects of anther and pollen development. *OsSTRL2* expression was detected only in tapetal cells and microspores, and knockout mutant resulted in male sterility mainly due to defects in anther wall and pollen exine formation (Zou et al., 2017). Several others in this category have been shown to regulate both vegetative and reproductive organ development. For example, *DNL1* (*Dwarf and Narrow Leafed 1*) encoding cellulose synthase like D4 is the major QTL regulating plant height and leaf width in rice. The mutant exhibits defects in anther dehiscence and pollen formation as well (Yoshikawa et al., 2013). Similarly, antisense transgenic plants of *OsTUB8* encoding β-tubulin exhibit shorter plant height as well as reduced seed set (Yang et al., 2007). Loss of function of *OsER2* (*ERECTA*) encoding phytosulfokine receptor led to reduced plant height and panicle size by affecting cell proliferation and growth in rice (Zhang et al., 2018). Furthermore, *SLG* (*Slender Grain*) from this group encodes BAHD acyltransferase and has been shown to regulate leaf angle, grain length, and plant height by modulating brassinosteroid levels (Liu et al., 2016). Interestingly, *OsSWEET11*, involved in sucrose transport and maintaining the sucrose concentration in the embryo-sac, also exhibited higher expression in EPs before heading (Ma et al., 2017).

Similarly, some of the genes exhibiting high-expressing genes in non-elongating peduncles as well as anther development have been experimentally shown to play role in grain filling and abiotic stress response. Among these, *OsMGD2* (monogalactosyldiacylglycerol synthase) affects plant height, anther development, and overall rice grain yield (Basnet et al., 2019). *SSG6* (*Substandard Starch Grain 6*) has previously been shown to regulate starch grain morphology and size in pollen and seeds (Matsushima et al., 2016). The *ssg6* mutant affected duration to heading, culm length, number of panicles, and seed weight. *OsSUT1* an important sucrose transporter is also upregulated after heading. Disruption of *OsSUT1* does not affect pollen maturation but their function gets impaired (Hirose et al., 2010), whereas *OsMST6* (Monfared et al., 2020) and *OsNHX1* (Almeida et al., 2017) regulate abiotic stress response. Interestingly, *OsCIPK23* (calcineurin B-like interacting protein kinase) has been implicated in mediating common signaling pathways between pollination and drought stress response (Yang et al., 2008).

The pivotal roles played by these genes in regulating anther development, plant height, grain filling, and abiotic stress response at the time of heading suggest that these as crucial candidates for alleviating the problem of panicle enclosure in male sterile lines and related agronomically important traits.

## Data Availability Statement

The original contributions presented in the study are publicly available. This data can be found here: https://www.ncbi.nlm.nih.gov/geo/query/acc.cgi?acc=GSE157727.

## Author Contributions

RS conceived and designed the study. MK performed all the experiments and the data analysis. MK and RS drafted the manuscript. CV, VC, KK, AP, and MKS participated in experiment design, sampling as well as implementation of the project. All authors read and approved the manuscript.

## Conflict of Interest

The authors declare that the research was conducted in the absence of any commercial or financial relationships that could be construed as a potential conflict of interest.

## Footnotes

1 http://rice.plantbiology.msu.edu/

2 https://stat.ethz.ch/R-manual/R-devel/library/stats/html/stats-package.html

3 https://cran.r-roject.org/web/packages/scatterplot3d/index.html

4 http://systemsbiology.cau.edu.cn/PlantGSEA/

5 https://mapman.gabipd.org/

6 http://qtaro.abr.affrc.go.jp/ogro

7 https://funricegenes.github.io/

## Supplementary Material

Supplementary Table 1 Summary statistics of RNA sequencing.

Supplementary Table 2A List of genes exhibiting higher expression in non-elongating peduncles (NP) compared to elongating peduncles (EP) in both Swarna and Pokkali.

Supplementary Table 2B List of genes exhibiting higher expression in elongating peduncles (EP) compared to non-elongating peduncles (NP) in both Swarna and Pokkali.

Supplementary Table 3A List of differentially expressed cellulose synthase and synthase like genes in rice peduncles.

Supplementary Table 3B List of differentially expressed lignin biosynthesis genes in rice peduncles.

Supplementary Table 3C List of differentially expressed carbohydrate metabolism and transport-related genes in rice peduncles.

Supplementary Table 3D List of differentially expressed hormone-related genes in rice peduncles.

Supplementary Table 3E List of differentially expressed transcription factor encoding genes in rice peduncles.

Supplementary Table 4 List of differentially expressed genes in rice peduncles overlapping with anther/pollen associated genes extracted from RiceAntherNet, funRiceGenes and OGRO databases.

Supplementary Table 5A Enriched MapMan pathways among upregulated genes in elongating peduncles (EP) of both Pokkali and Swarna plants.

Supplementary Table 5B Enriched MapMan pathways among the genes exclusively upregulated in elongating peduncles (EP) of Pokkali.

Supplementary Table 5C Enriched MapMan pathways among exclusively upregulated genes in elongating peduncles (EP) of Swarna.

Supplementary Table 5D Enriched MapMan pathways among upregulated genes in non-elongating (NP) peduncles of both Pokkali and Swarna plants.

Supplementary Table 5E Enriched MapMan pathways among exclusively upregulated genes in non-elongating (NP) peduncles of Pokkali.

Supplementary Table 5F Enriched MapMan pathways among exclusively upregulated genes in non-elongating (NP) peduncles of Swarna.
